# Epidermal growth factor receptor-targeted sonoporation with microbubbles enhances therapeutic efficacy in a squamous cell carcinoma model

**DOI:** 10.1371/journal.pone.0185293

**Published:** 2017-09-22

**Authors:** Fumika Hirabayashi, Kenjiro Iwanaga, Toshinori Okinaga, Osamu Takahashi, Wataru Ariyoshi, Ryo Suzuki, Mutsumi Sugii, Kazuo Maruyama, Kazuhiro Tominaga, Tatsuji Nishihara

**Affiliations:** 1 Division of Infections and Molecular Biology, Department of Health Promotion, Kyushu Dental University, Kitakyushu, Japan; 2 Division of Oral and Maxillofacial Surgery, Department of Science of Physical Functions, Kyushu Dental University, Kitakyushu, Japan; 3 Laboratory of Drug Delivery System, Faculty of Pharma-Sciences, Teikyo University, Tokyo, Japan; Argonne National Laboratory, UNITED STATES

## Abstract

Sonoporation is a drug and gene delivery system using ultrasonication that allows the intracellular delivery of foreign molecules that cannot enter cells under normal conditions. We previously reported that sonoporation with microbubbles (MBs) could achieve effective intracellular drug delivery to human gingival squamous carcinoma Ca9-22 cells. In this study, we developed anti-epidermal growth factor receptor (EGFR) antibody-conjugated MBs (EGFR-MBs) and evaluated their capacity to enhance anti-cancer drug toxicity *in vitro* and *in vivo*. We first assessed the effect of sonoporation with EGFR-MBs on Ca9-22 cells by the WST-8 assay, flow cytometry and Hoechst’s staining *in vitro*. Sonoporation and EGFR-MB had a strong cytotoxic effect on Ca9-22 cells with low-dose bleomycin. Furthermore, bleomycin delivery using sonoporation with EGFR-MBs remarkably increased the number of apoptotic cells. We next examined the effect of EGFR-MBs in a murine squamous cell carcinoma model. Bleomycin delivery by sonoporation with EGFR-MBs exhibited remarkable antitumor activity. Together, our results show that EGFR-MBs and ultrasound treatment increases the efficacy and specificity of intracellular drug uptake, suggesting this could be a novel drug-targeting modality for oral squamous cell carcinoma chemotherapy treatment.

## Introduction

Medical ultrasound (US) imaging has made remarkable progress in the development of clinical US technology. Due to its safety and convenience, US imaging has become an indispensable tool for various medical and scientific fields. Recently, ultrasonication has been proposed as an effective drug and gene delivery system for treatment [1]. US creates transient permeability of the cell membrane that enables foreign molecules to enter cells, a process known as sonoporation [2,3]. The effect of sonoporation is further enhanced by the presence of microbubble (MB) echo contrast agents [4–6]. We previously reported that these techniques can be used to load anti-proliferating agents and plasmid DNA into cells [7].

Epidermal growth factor receptor (EGFR) is a 170 kDa receptor tyrosine kinase consisting of an extracellular ligand binding domain in the amino terminal followed by a hydrophobic transmembrane domain [8]. EGFR is involved in cell proliferation, migration, invasion and survival [9]. EGFR overexpression is found in a number of human malignancies, including breast, ovary, bladder, kidney, pancreas and oral cancers [10]. Therefore, EGFR is an attractive target for antitumor treatment [11,12]. We previously demonstrated that sonoporation with MBs and an anti-EGFR antibody could enable effective delivery of the anticancer drug bleomycin (BLM) *in vitro* [13]. However, we used MBs and an anti-EGFR antibody suspension for sonoporation, and could not determine whether the MBs and anti-EGFR antibody specifically bound cells. Therefore, further investigation is necessary to develop an effective and specific drug delivery system for chemotherapy. Receptor-based targeting is a promising approach for the development of targeted cancer therapy, and liposomes are good candidates for drug delivery [14,15].

In this study, we developed an anti-EGFR antibody-conjugated MB (EGFR-MB) as a targeting agent and investigated the efficacy of BLM delivery using sonoporation with EGFR-MBs *in vitro* and *in vivo* in a squamous cell carcinoma model. Our results show that EGFR-MBs are an effective and specific drug delivery system against cancer cells.

## Material and methods

### Cell lines, reagents and antibody

Ca9-22 cells derived from human gingival squamous cell carcinoma were provided from the Japanese Collection of Research Bioresources (JCRB) (Osaka, Japan) and cultured in RPMI 1640 medium (Nacalai Tesque, Kyoto, Japan) supplemented with 10% heat-inactivated fetal bovine serum (FBS), penicillin (100 U/mL) (Nacalai Tesque) and streptomycin (100 mg) (Nacalai Tesque) at 37°C in a humidified atmosphere with 5% CO_2_. BLM was purchased from LKT Laboratories (St. Paul, MN, USA). Anti-EGFR antibody was prepared as described previously [13]. Briefly, culture supernatants from the 528 hybridomas (ATCC; TKG 0555, Manassas, VA, USA) were collected and fractionated with 60% ammonium sulfate to prepare the anti-EGFR antibody, and the final pellet, which contained the crude anti-EGFR antibody, was dissolved in phosphate-buffered saline (PBS). The crude anti-EGFR antibody was then purified using a Nab Protein A plus Spin Kit (PIERCE, Rockford, IL, USA). Control IgG from mouse serum was purchased from SIGMA ALDRICH (St. Louis, MO, USA).

### Preparation of antibody-modified lipid (DSPE-PEG (2k)-Ab)

We dissolved 3-(N-succinimidyloxyglutaryl) aminopropyl, polyethyleneglycol 2000-carbamoyl distearoyl-phosphoethanolamine (DSPE-PEG (2k)-NHS, SUNBRIGHT DSPE-020GS; NOF Corporation, Tokyo, Japan) (0.123 mg) in chloroform. The lipid solution was evaporated to make a lipid film in a glass tube by chloroform removal. Then, the antibody solution (0.125 mg/mL, 0.56 mL) in PBS (pH 7.4) was added to the lipid film. The lipid film was rehydrated with antibody solution to conjugate the antibody to DSPE-PEG (2k)-NHS. The sample was incubated at 60°C for 5 min, and then at room temperature for 1 h to obtain the antibody-conjugated PEG-lipid (DSPE-PEG (2k)-Ab).

### Preparation of antibody-conjugated MBs

We dissolved 1, 2-distearoyl-sn-glycero-3-phosphocholine (DSPC, COATSOME MC-8080; NOF Corporation) and 1, 2-distearoyl-sn-glycero-3-phosphoethanolamine (DSPE-PEG (5k)-OMe, SUNBRIGHT DSPE-050CN; NOF Corporation) in chloroform, and the lipid solution was evaporated to make a lipid film in a glass tube by chloroform removal. The lipid film was then rehydrated with PBS (pH 7.4) (0.462 mL). This lipid suspension (0.462 mL) of DSPC and DSPE-PEG (5k)-OMe, the suspension (0.493 mL) of DSPE-PEG (2k)-Ab (antibody-modified) or DSPE-PEG (2k)-OMe (control) and propylene glycol (0.045 mL) were mixed in the glass vial (2 mL vial). The head space of the vial was filled with perfluoropropane (C3F8) (Takachiho Chemical Industrial CO., LTD., Tokyo, Japan). The C3F8-filled vial was shaken for 45 s with VIALMIX (Lantheus Medical Imaging, Billerica, MA, USA), and the vial was cooled on ice for 5 min. To remove large bubbles, the vial was placed upside down for 15 min. Smaller bubbles were taken from the lower layer in the vial with a 24G needle attached to a syringe. The mean size and number of the MBs were measured with a Multisizer3 (Beckman Coulter, Brea, CA, USA). MBs were labeled with the fluorescence probe 3, 3'-dioctadecyloxacarbocyanine perchlorate (0.53 mg) (DiO, Thermo Fisher Scientific, Waltham, MA, USA), and we prepared the lipid film including DiO (1.6 mg/total lipid, 60 mg).

### Immunofluorescence analysis

The day before the experiments, Ca9-22 cells (1.5×10^6^ cells/well) were incubated with DiO-labeled MBs, IgG-MBs or EGFR-MBs for 5 min at 37°C. To confirm the binding of EGFR-MBs to Ca9-22 cells, the cells were washed and collected, and fluorescence intensities were measured by flow cytometry (EPICS XL; Beckman Coulter).

### *In vitro* BLM sonoporation

*In vitro* BLM delivery into Ca9-22 cells with EGFR-MBs and US exposure was performed using previously described methods [7, 13]. Briefly, cultured cells were harvested by trypsinization, washed once in PBS and resuspended at 1.5×10^6^ cells/600 μL of serum-free RPMI1640 in a 48-well plate. MBs, IgG-MBs or EGFR-MBs were added to the cell suspension, mixed and incubated for 5 min at 37°C. After incubating MBs with the cells, the BLM solution was added to the cell suspension at a final concentration of 5 μg/mL. Then, Ca9-22 cells were immediately exposed to US (frequency, 1 MHz; duty cycle, 10%; output intensity, 1.0 W/cm^2^) for 20 s at room temperature using a ultrasonication probe placed in each well, and then washed twice with PBS. US was generated using a Sonitron 2000 sonicator (Rich Mar Inc., Inola, OK, USA).

### Cell proliferation quantification

Cell proliferation was determined using WST-8 assays (Dojindo Laboratories Co., Kumamoto, Japan). After treating Ca9-22 cells, the cells were washed in PBS, seeded in flat-bottomed 96-well plates at a concentration of 2.0×10^4^ cells/mL and cultured in RPMI 1640 containing 5% FBS. After 48 h, 10 μL of WST-8 reagent was added to each well and incubated for 4 h. Absorbance at 450 nm was measured using a Multiskan JX microplate reader (Thermo Fisher Scientific).

### Apoptosis assay

After Ca9-22 cells were treated as above, the cells were collected, washed in PBS and treated with propidium iodide (PI). DNA contents were analyzed using an EPICS XL (Beckman Coulter). For Annexin V/PI staining, the treated cells were washed once with PBS and treated with the Annexin-V-FLUOS Staining kit (Roche Applied Science, Indianapolis, IN, USA) as previously described [7, 13]. After incubation, treated cells were analyzed using an EPICS XL (Beckman Coulter), and apoptotic cell death was detected using the florescent nuclear dye Hoechst 33258 (Dojindo Laboratories Co.) and fluorescence microscopy.

### Mouse tumor xenograft model

Male 6-week-old KSN/slc nude mice weighting 20-25g were purchased from SLC (Shizuoka, Japan). The squamous cell carcinoma xenograft model was established as described previously [7]. Briefly, Ca9-22 cells (1×10^6^ in 0.2 mL serum-free RPMI1640) were subcutaneously injected into the backs of the mice. Tumor size was measured daily, and tumor volume was calculated by the formula: volume (mm^3^) = length (mm) × width^2^ (mm^2^)/2. This study was carried out in strict accordance with the recommendations in the Guide for the Care and Use of Laboratory Animals of the National Institutes of Health. The protocol was approved by the Kyushu Dental University Experimental Animal Care and Use Committee (Permit Numbers: 16–025). A completed ARRIVE guidelines checklist is included in Checklist S1 File.

### *In vivo* BLM sonoporation

KSN/slc nude mice bearing 50–60 mm^3^ Ca9-22 tumors were randomly assigned into four groups: control, BLM injection, BLM injection and MB sonoporation, and BLM injection and EGFR-MB sonoporation. Mice were anesthetized and MBs or EGFR-MBs (2.5×10^7^) were directly injected into the tumor region with a 27-gauge needle. At 5 min after MB injection, the mice were intravenously injected with BLM via the tail vein at a final concentration of 0.4 mg/mL. Then, the tumor region was covered with an ultrasonication conducting gel and immediately exposed to US (frequency, 1 MHz; duty, 50%; output intensity, 2.0 W/cm^2^) twice for 1 min. Tumor size and body mass of treated mice were measured every other day beginning on day 0 of sonoporation treatment.

### Histochemical analysis

After 4 weeks, the mice were sacrificed, and sections of tumor tissues from each group were prepared. The TUNEL assay was performed using a Tumor TACS apoptosis detection kit (Trevigen, Inc. Gaithersburg, MD, USA) according to the manufacturer’s instructions to detect nuclear DNA fragmentation.

### Statistical analysis

Values are expressed as means ± standard divisions of the mean. Differences between groups were assessed by one-way analysis of variance (ANOVA) with a suitable post-test.

## Results

### Specific binding of EGFR-MBs to Ca9-22 cells

**Fig 1:**
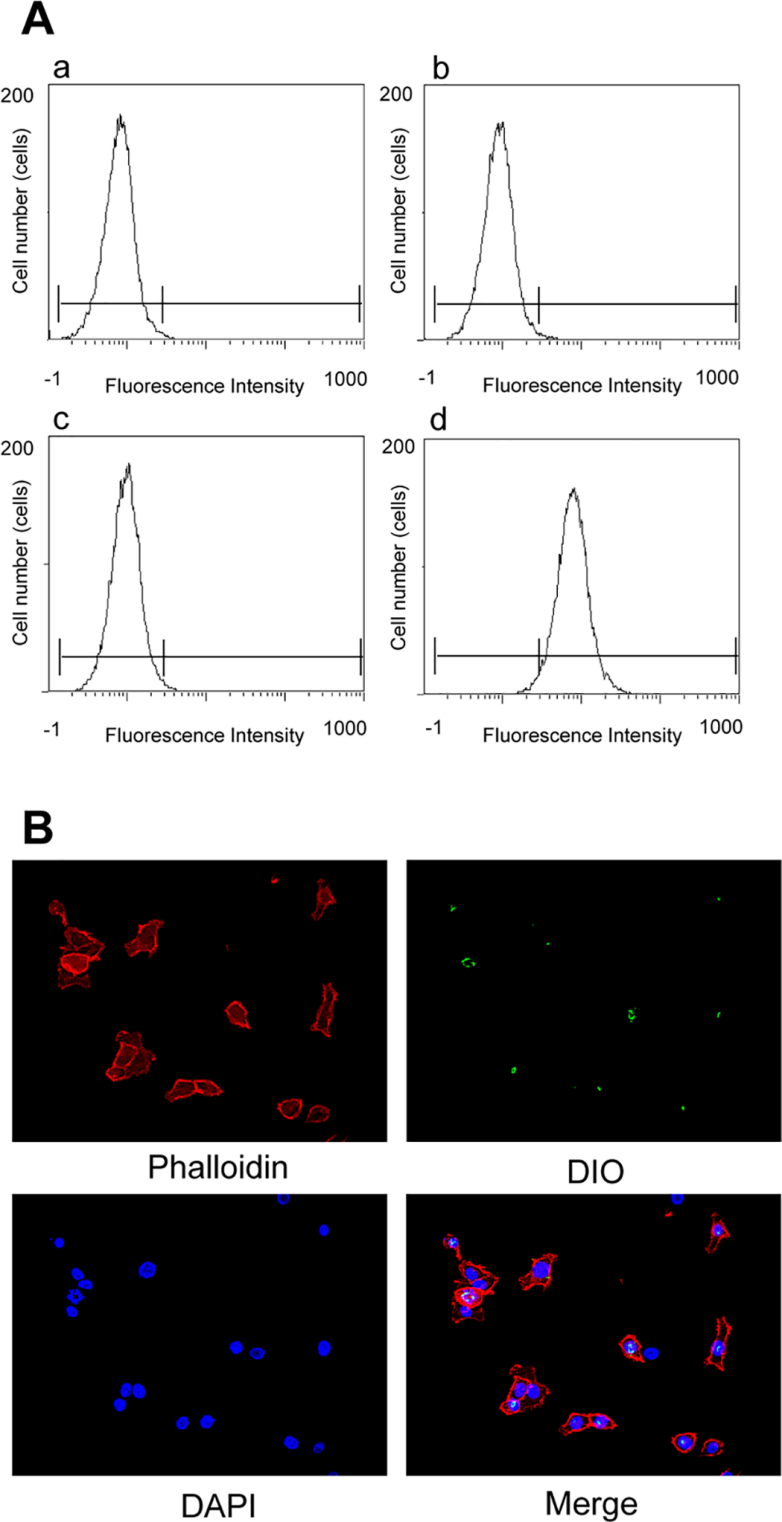
Specific binding of EGFR-MBs to EGFR on Ca9-22 cells. (A) Fluorescence intensity was measured by flow cytometry; untreated Ca9-22 cells (a), Ca9-22 cells treated with DIO-labeled MBs (b), DIO-labeled IgG-MBs (c), and DIO-labeled EGFR-MBs (d). (B) Ca9-22 cells were incubated with EGFR-MBs for 30 min at 37°C, fixed and stained as indicated. Stained cells were examined using a fluorescence microscope.

We first confirmed the specific binding of EGFR-MBs to EGFR on Ca9-22 cells by flow cytometry and immunofluorescence staining. Flow cytometric analysis demonstrated that EGFR-MBs specifically bound to EGFR on Ca9-22 cells (Fig 1A). We also confirmed that MBs and/or control IgG-conjugated MBs did not bind to EGFR on Ca9-22 cells. Immunofluorescence staining also confirmed specific binding of EGFR-MBs to EGFR on Ca9-22 cells (Fig 1B).

### *In vitro* growth inhibition of Ca9-22 cells after delivery of BLM by sonoporation

**Fig 2:**
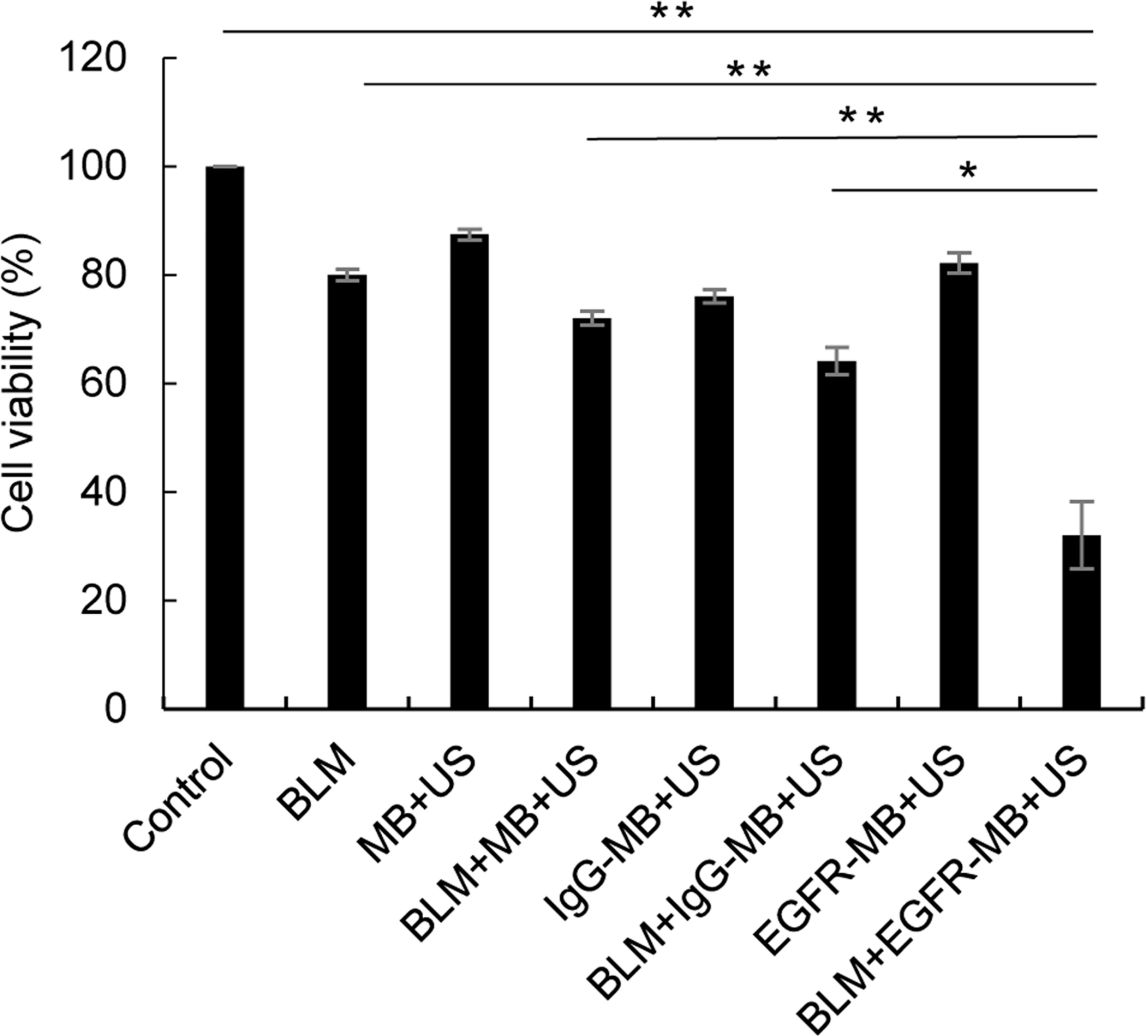
*In vitro* growth inhibition of Ca9-22 cells after BLM delivery. Ca9-22 cells were treated with BLM by sonoporation using microbubbles (MB+US), IgG-conjugated MBs (IgG-MB+US) or anti-EGFR antibody conjugated MBs (EGFR-MB+US) as indicated. After 48 h incubation, cell proliferation was measured using WST-8 assays. **P<0.001, *P<0.05.

We next examined the effects on cell proliferation after delivery of BLM by sonoporation using EGFR-MBs. Although, sonoporation with MBs or IgG-MBs enhanced the cell killing effect of low-dose of BLM, BLM delivery using sonoporation with EGFR-MBs was significantly more toxic to the cells compared with the other groups (Fig 2).

### Apoptosis in Ca9-22 cells after BLM delivery by sonoporation *in vitro*

**Fig 3:**
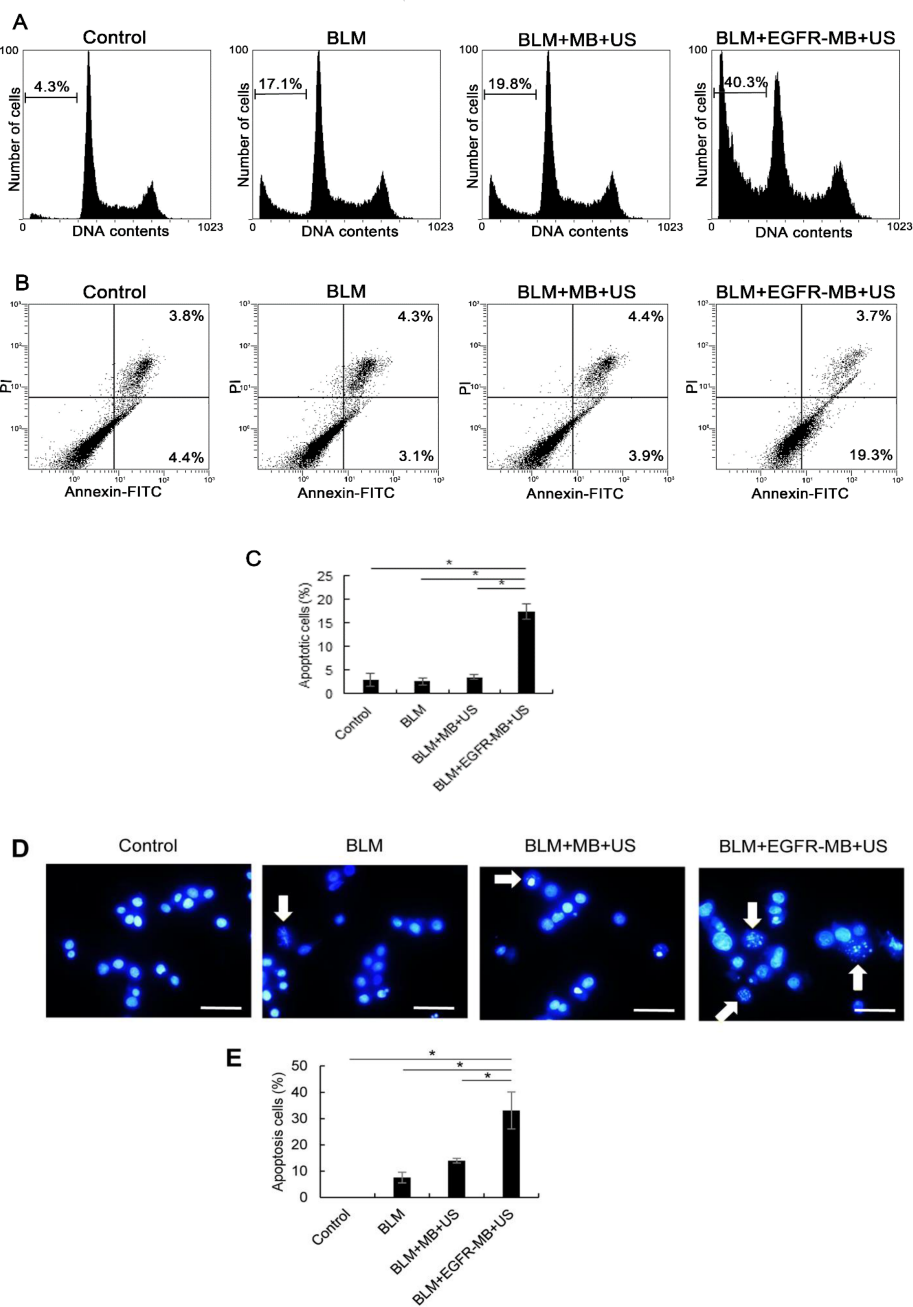
Apoptosis in Ca9-22 cells after BLM delivery *in vitro*. (A) Cell cycle distribution analyses by flow cytometry in the no treatment, BLM alone, BLM + sonoporation + MBs, or BLM + sonoporation + anti-EGFR antibody-conjugated MBs groups. The percentage of cells in the sub-G1 phase is indicated. (B) Apoptosis analysis by flow cytometry in the no treatment, BLM alone, BLM + sonoporation + MBs, or BLM + sonoporation + anti-EGFR antibody-conjugated MBs groups. (C) Percentages of apoptotic cells. *P<0.001 (D) Hoechst staining was performed to observe morphological changes in Ca9-22 cells after 48 h BLM delivery treatment. Cells were exposed to no treatment, BLM alone, BLM + sonoporation + MBs, or BLM + sonoporation + anti-EGFR antibody-conjugated MBs. Apoptotic cells (arrows) exhibited characteristic chromatin condensation under fluorescence microscopy. Bar: 20 μm. (E) Percentages of apoptotic cells. *P<0.01.

To examine whether BLM delivery by sonoporation with EGFR-MBs exhibited cytotoxicity in Ca9-22 cells through apoptosis, PI staining was undertaken to detect hypodiploid DNA. Ca9-22 cells treated by sonoporation with BLM (5 μg/mL) and EGFR-MBs showed increased numbers of cells in subG1 phase (40.3%) compared with cells treated with BLM or BLM and MBs (17.1% and 19.8%, respectively) (Fig 3A). To quantify apoptosis, treated cells were analyzed by the Annexin V-FITC/PI cell apoptosis assay combined with flow cytometry. As shown in Fig 3B and 3C, delivery of BLM by sonoporation with EGFR-MBs show antitumor activities in Ca9-22 cells, mainly through the induction of apoptotic cell death. We also examined apoptotic nuclei in BLM-delivered Ca9-22 cells with Hoechst staining (Fig 3D). Morphological evidence of apoptosis was detected as chromatin condensation and nuclear fragmentation following 48 h treatment of BLM delivery by sonoporation in the presence of EGFR-MBs in Ca9-22 cells. Taken together, these results indicated that intracellular low-dose BLM delivery by sonoporation with EGFR-MBs *in vitro* induced apoptosis in Ca9-22 cells.

### *In vivo* growth inhibition of Ca9-22 xenografts after BLM delivery by sonoporation

**Fig 4:**
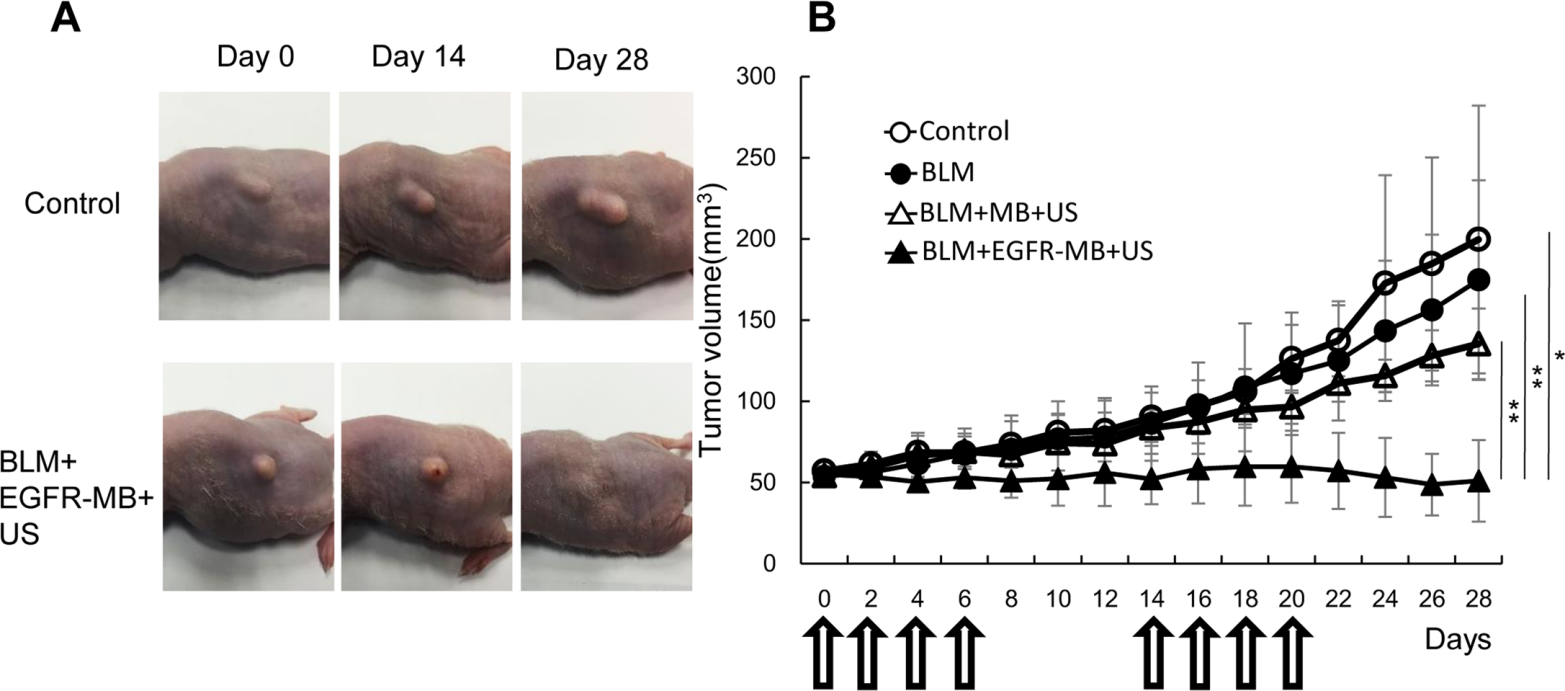
Growth inhibition of Ca9-22 xenografts after BLM delivery. KSN/slc nude mice were injected with Ca9-22 cells and divided into four experimental groups: control (n = 4), BLM injection (n = 4), sonoporation with MBs and BLM injection (n = 4) and sonoporation with EGFR-MBs and BLM injection (n = 4). (A) Images of mice from the different groups on days 0, 14 and 28. (B) Tumor volumes of the different treatment groups. Arrows indicate the days mice received treatments. Data are expressed as the means ± s.e.; **P<0.01, *P<0.05.

Finally, we examined the effect of BLM delivery by sonoporation with EGFR-MBs on *in vivo* tumor growth in KSN/slc nude mice. On day 0, BLM (40 μg) was injected in the tail vein and EGFR-MBs were intratumorally injected. Tumor growth was observed for 28 d in Ca9-22-bearing mice. The growth curves of Ca9-22 tumors are shown in Fig 4B. Tumor volume increased in the Ca9-22-inoculated control group and in the BLM-injected group, whereas the group injected with BLM in the presence of MBs showed smaller tumors (Fig 4A and 4B). Significantly higher anti-tumor effects against Ca9-22 cells were observed in mice treated with BLM compared with the other groups. There were no significant changes in body weight between the four groups (data not shown).

### Apoptosis in Ca9-22 cells after BLM delivery by sonoporation *in vivo*

**Fig 5:**
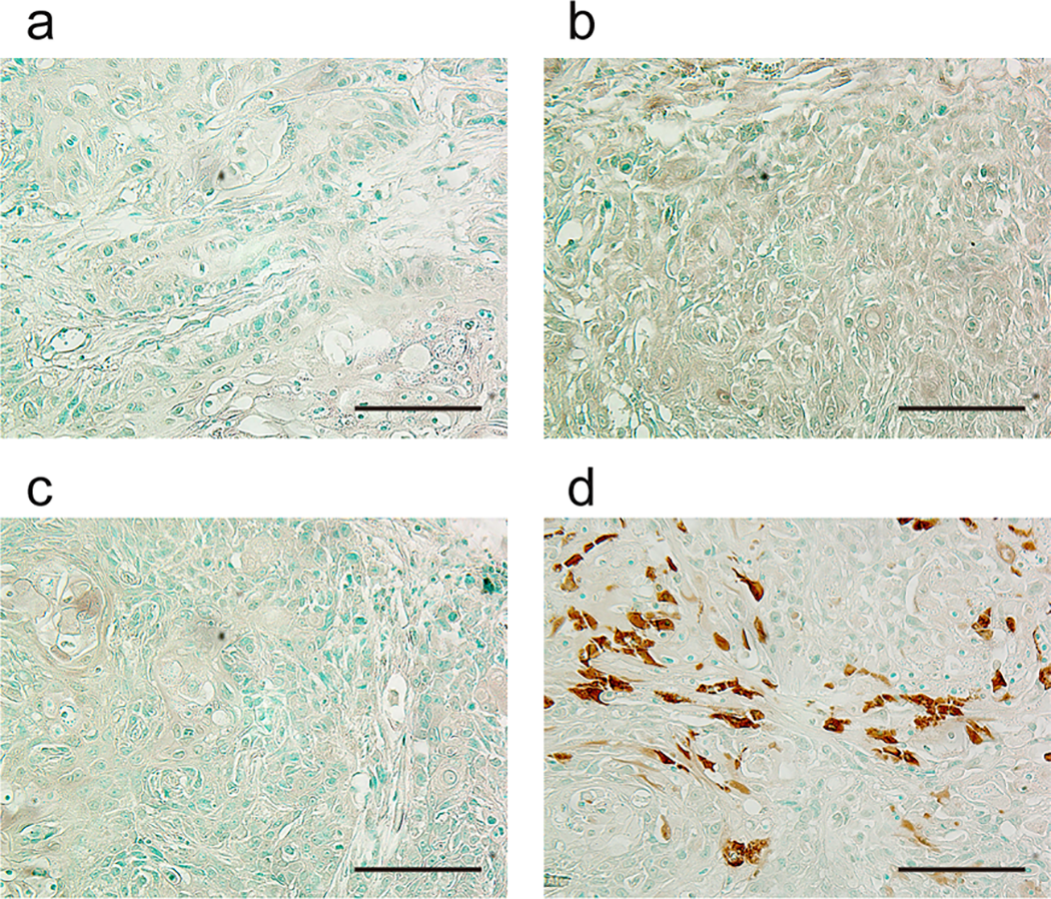
TUNEL analysis of xenografts after *in vivo* sonoporation with BLM. Ca9-22 xenograft–bearing mice were exposed to sonoporation *in vivo*. (a) control, (b) BLM injection, (c) sonoporation with MBs and BLM injection, (d) sonoporation with EGFR-MBs and BLM injection. TUNEL signal was visualized by diaminobezidine (DAB, brown) and cell nuclei were counterstained by methyl green. Magnification, 400×; bar, 100 μm.

TUNEL staining clearly showed apoptosis in xenograft tumor samples from mice treated by sonoporation in the presence of BLM and EGFR-MBs (Fig 5).

## Discussion

We investigated the therapeutic effect of EGFR-MBs in oral squamous cell carcinoma by administration of BLM and EGFR-MBs coupled with US. Repeated applications of high-dose antitumor drugs are commonly needed in systematic chemotherapy, which results in severe side effects. Thus, it is important to develop easy, safe, effective and minimally-invasive techniques for anticancer drug delivery into tumor cells. Many researchers have tried to establish new drug delivery systems that combine high drug delivery efficiency with reduced invasiveness. To this end, we and others have developed the application of non-thermal US energy for drug-targeting or drug-controlled release [16]. Several groups have reported that sonoporation with MBs could be a feasible method to deliver BLM to human cancer cell lines *in vitro* and *in vivo* [7,17]. Additionally, we demonstrated the growth inhibitory effect of BLM delivered by sonoporation with anti-EGFR antibody *in vitro* [13]. However, we found no evidence of the binding specificity of MBs and an anti-EGFR antibody. This study demonstrated that the use of EGFR-MBs combined with US exposure enhanced BLM delivery in an oral squamous cell carcinoma model.

The human gingival squamous cell carcinoma cell line Ca9-22 shows cell surface EGFR overexpression [18]. In this study, we used EGFR-MBs that were recently developed in the Faculty of Pharma-sciences, Teikyo University and assessed their specific binding to Ca9-22 cells. Flow cytometric analysis and immunofluorescence staining revealed that the EGFR-MBs bound to EGFR on Ca9-22 cells (Fig 1).

A safe and effective therapeutic approach involving sonoporation together with chemotherapeutic agents for cancer treatment has the advantage of reducing drug dose to avoid side effects in clinical practice. BLM displays cytotoxic activity due to generating DNA breaks. [19]. BLM induces apoptosis via DNA strand breaks through an oxygen- and metal ion-dependent process in mammalian cells, which are seen as chromosomal gaps, deletions and DNA fragmentation. [20,21]. Furthermore, it has been shown *in vitro* that less than 0.1% BLM in the cell culture medium is extremely toxic to normal cells. [22]. In this study, we demonstrated that the combined use of low-concentration of BLM and EGFR-MBs with US exposure could deliver an effective dose of the chemotherapeutic agent into cells. We achieved effective intracellular BLM delivery *in vitro* and observed that cell viability decreased by 70% in the presence of EGFR-MBs (Fig 2). Furthermore, apoptosis after sonoporation using low-concentration BLM (5 μg/mL) and EGFR-MBs was significantly higher compared with the other treatment groups (Fig 3).

Sonoporation allows localized temporary permeabilization (i.e. pore formation) of the cell membrane by ultrasonication, which can be followed by the entry of foreign molecules into cells *in vitro* and *in vivo* [23,24]. Many researchers have focused on establishing the sonoporation system as an effective non-viral drug delivery method. Sonoporation strategies have also been developed with various types of MBs for targeted delivery [25,26]. Liao et al. demonstrated that the combination of EGFR-targeting MBs and US exposure effectively eliminates tumor cells *in vivo* [27]. Dewitte et al. reported effective cancer immunotherapy using US and mRNA-loaded MBs for dendritic cell delivery [28]. Previous findings on the enhanced effect of sonoporation provided by targeted MBs led us to investigate its *in vivo* therapeutic potential with BLM.

In this study, we used unique MBs that consisted of liposomes containing polyethylene glycol (PEG) chains at their surface to prevent recognition by the reticulo-endothelial system, generating so-called stealth liposomes [29,30]. A PEG-liposome of less than 200 μm results in the passive accumulation of stealth liposomes in the tumor vasculature from enhanced permeability and retention [31,32]. We also previously used nano-sized bubble liposomes as a sonoporation agent for drug delivery. However, the application of nano-bubble liposomes is hindered by problems associated with their stability in solution due to particle size; thus, we could not achieve effective drug delivery (data not shown). Therefore, we developed and examined a new micro-sized liposome (1–10 μm) in this study. Although EGFR-MBs had difficulty permeating vasculature, they showed good stability and long circulation once they enter tissues by sonoporation. In this study, EGFR-MBs were directly injected into the tumor region, and BLM was injected via the tail vein. The results obtained in this study can be explained by the remarkable increase of BLM uptake by targeted sonoporation following EGFR-MBs specific binding to tumor cells. Our results showed that sonoporation with EGFR-MBs showed a greater antitumor effect compared with sonoporation with MBs (Figs 4 and 5). These findings indicated that sonoporation with EGFR-MBs remarkably enhanced BLM cytotoxicity in Ca9-22 cells *in vitro* and *in vivo*. Taken together, this application may be useful for drug delivery in solid tumors.

Several conjugation techniques exist to couple ligands to microbubbles. In this study, we attached the targeting ligand, EGFR antibody, using PEG for targeting strategy. However, PEGylation reduces complement activation, which induce adaptive immune response with repeated usage [33]. In this case, for the success of targeted immunotherapy, the homogeneous of tumor-associated antigen is critical for development our drug delivery system. Furthermore, foreign proteins can cause an immune response. Antibodies have clinical limitations under immune response, on using antibodies targeting delivery in humans. We have to design antibody for adapting to human in each treatment. EGFR plays a crucial role in malignant cell growth, proliferation and survival of cancer cells. And also, EGFR is widely observed in several malignant. However, it is unclear how the mutational status, gene copy number and EGFR overexprresion impacts signaling pathway in cancer [34, 35]. We should identify a biomarker that can expect anti-EGFR therapy response, Further clinical investigation is needed to validate the potential of sonoporation with EGFR-MBs.

## Conclusions

We have demonstrated that sonoporation with EGFR-MBs is an effective targeted drug delivery system for oral squamous cell carcinoma. Our results show that treatment with EGFR-MBs, especially with micro-sized liposomes and US make it possible to more effectively and specifically administer anticancer drugs into cells. Our study demonstrated that EGFR-targeted sonoporation with MBs may hold promise as new effective therapies for oral squamous cell carcinoma.

## Supporting information

S1 File ARRIVE guidelines checklist. (DOCX)
